# Muscle pain in a woman with congenital adrenal hyperplasia due to 21-hydroxylase deficiency resolved with testosterone therapy. A case report with 10 years of follow-up

**DOI:** 10.3389/fendo.2026.1757725

**Published:** 2026-02-24

**Authors:** Joanna Hubska, Paulina Jaszczuk, Joanna Betlejewska, Natalia Bylińska, Małgorzata Bobrowicz, Beata Rak-Makowska, Urszula Ambroziak

**Affiliations:** 1 Department of Internal Medicine and Endocrinology, Medical University of Warsaw, Warsaw, Poland; 2 Doctoral School of Medical University of Warsaw, Medical University of Warsaw, Warsaw, Poland; 3 Student Scientific Club “Endocrinus” Affiliated to the Department of Internal Medicine and Endocrinology, Medical University of Warsaw, Warsaw, Poland; 4 Laboratory of Experimental Medicine, Medical University of Warsaw, Warsaw, Poland; 5 Department of Biochemistry and Molecular Biology, Centre of Translational Research, Centre of Postgraduate Medical Education, Warsaw, Poland

**Keywords:** 21-hydroxylase deficiency, androgen deficiency, CAH, congenital adrenal hyperplasia, glucocorticoid overtreatment, muscle weakness, testosterone therapy

## Abstract

Congenital adrenal hyperplasia (CAH) due to 21-hydroxylase deficiency (21OHD) requires lifelong glucocorticoid (GC) and mineralocorticoid therapy to prevent adrenal crises and control androgen excess. However, chronic GC overtreatment may result in sustained suppression of adrenal androgens — an underrecognized complication with significant implications for women. Androgens contribute to muscle function, mood regulation, and sexual health, yet symptoms of deficiency are easily misattributed. We report a 32-year-old woman with classical salt-wasting CAH who presented with severe muscle pain, weakness, reduced libido, and depressive symptoms. Laboratory results revealed complete suppression of adrenocorticotrophin hormone, dehydroepiandrosterone sulfate, and testosterone, with normal creatine kinase, electrolytes, metabolic and rheumatologic parameters. Extensive neuromuscular evaluation was unremarkable. Childhood medical records confirmed persistent suppression of adrenal androgens from infancy, indicating long-standing GC overtreatment as the most likely cause. Because GC dose reduction was poorly tolerated and no alternative explanation for her symptoms was identified, low-dose intramuscular testosterone (50 mg every 4–8 weeks) was introduced as compassionate therapy and subsequently stabilized at 25 mg every 4 weeks. Within three months, the patient reported substantial improvement in muscle pain, strength, libido, and mood. Over ten years of follow-up, testosterone therapy remained well tolerated, with no side effects such as virilization, erythrocytosis, hepatotoxicity, dyslipidemia, or menstrual disturbances. Bone density and trabecular microarchitecture remained stable. This case demonstrates that chronic GC overtreatment may lead to profound androgen deficiency in women with CAH, which can manifest as debilitating musculoskeletal and neurobehavioral symptoms. The patient’s sustained clinical improvement underscores the physiological importance of androgens in female health and supports consideration of individualized, low-dose testosterone replacement in carefully selected cases. Recognition and targeted treatment of androgen deficiency should form part of long-term CAH management. To our knowledge, this is the first report describing the resolution of chronic myalgia after testosterone therapy in a woman with CAH and complete adrenal androgen suppression.

## Introduction

Congenital adrenal hyperplasia (CAH) refers to a group of autosomal recessive disorders caused by defects in enzymes involved in adrenal steroidogenesis, leading to impaired cortisol biosynthesis. Among these, 21-hydroxylase deficiency (21OHD)—caused by pathogenic variants in the *CYP21A2* gene—accounts for over 90–95% of cases. Chronic cortisol deficiency leads to elevation of adrenocorticotropic hormone (ACTH) through the loss of negative feedback loop, resulting in adrenal hyperplasia and overproduction of androgenic precursors such as 17-hydroxyprogesterone (17-OHP) and androstenedione (1–3).

Clinically, 21OHD manifests across a spectrum ranging from the classical salt-wasting (SW) form, with life-threatening neonatal adrenal crisis, to the simple virilizing (SV) and non-classical forms characterized by milder symptoms. The SW phenotype, as in the present case, results from near-complete loss of enzyme activity and combined deficiency of cortisol and aldosterone. Early diagnosis and lifelong hormone replacement therapy are therefore essential for survival (4).

Here, we report the case of a 32-year-old woman with classical SW 21OHD who developed severe muscle pain, weakness, and depressive symptoms associated with complete suppression of adrenal androgens secondary to chronic GC overtreatment during childhood. To the best of our knowledge, this is the first reported case demonstrating improvement in muscle pain following testosterone therapy in a female patient with CAH.

## Case description

A 32-year-old female with classical SW CAH due to 21OHD was admitted to the endocrine department for evaluation of severe muscle pain, weakness, decreased libido, and depression. Her chronic regimen included prednisolone 10 mg/day (administered in two daily doses) and fludrocortisone 0.1 mg/day. She had also taken zolpidem for insomnia and used metamizole and paracetamol regularly for muscle pain in her arms and legs, reporting increasing dependence on these medications over the past three years.

On examination, she presented with weight gain, facial plethora, and easy bruising suggestive of GC excess. Menstrual cycles were regular, and no signs of hyperandrogenism were noted. A previous attempt to reduce prednisolone to 7.5 mg/day three years earlier led to deterioration, with marked fatigue and reduced energy, prompting return to the 10 mg/day dose.

On admission, laboratory tests revealed complete suppression of ACTH, dehydroepiandrosterone sulfate (DHEAS), and testosterone. Electrolytes, creatinine kinase (CK), lactate dehydrogenase (LDH), alanine aminotransferase (ALT), and aspartate aminotransferase (AST) were within normal ranges (**Table 1**). A comprehensive diagnostic evaluation of muscle symptoms was performed, including electromyography, nerve conduction studies, lactate curve test— all of which were unremarkable. Comprehensive rheumatologic serology, including RF, ANA, anti-dsDNA, ENA panel (U1-RNP, Sm, SSA, SSB, Scl-70, centromere B, Jo-1), and MPO-ANCA, was entirely within normal limits, providing no evidence of an underlying autoimmune rheumatologic disorder. Brain MRI and abdominal ultrasound were normal. Dual-energy X-ray absorptiometry (DXA) showed normal bone mineral density (BMD) of the lumbar spine (L1–L4: 1.015 g/cm², T-score –0.3, Z-score -0.3) and femoral neck (BMD 0.784 g/cm², T-score –0.6, Z-score -0.4), with no evidence of vertebral deformities on lateral spine assessment. Whole-body DXA revealed normal total skeletal BMD (1.29 g/cm², T-score +2.2, Z-score +2.4) and body composition characterized by a high lean mass (lean tissue 40.8 kg, fat mass 9.8 kg; body fat 18.5%), all within normal ranges for adult females.

**Table 1:** Laboratory test results on admission, two years after, and ten years after testosterone initiation.

Parameter	Unit	Admission day	2 years after testosterone initiation	5 years after testosterone initiation	6 years after testosterone initiation	8 years after testosterone initiation	10 years after testosterone initiation	Reference range
General laboratory results								
ALT	U/l	12	10	9	–	9.5	14	7–35
AST	U/l	20	25	–	–	20.2	21	7–35
ALP	IU	48	37	–	–	–	–	35–104
CK	IU	69	–	–	–	–	–	26–140
Creatinine	mg/dl	0.93	0.86	0.88	0.86	–	0.72	0.5–1.1
eGFR (CKD-EPI)	ml/min/1.73 m²	98	>90	>90	>90	–	104	≥ 90
Fasting glucose	mg/dl	73	68	60	–	68.1	79	60–99
HbA1c%	%	4.8	4.9	–	–	–	5.2	(4.5-6.5)
Hemoglobin	g/dl	13.9	13.58	13.7	13.1	12.8	13.9	12–16
Hematocrit	%	43.1	41.2	43	42.1	40.1	43.6	37-47
Red blood count	ug/dl	4.93	4.56	4.82	4.52	4.21	4.97	3.8-5.2 x10^6^
Potassium	mmol/l	4.16	4.03	4.11	4.86	3.99	4.2	3.5–5.0
Sodium	mmol/l	136.8	142.5	140	139	142	140	135–145
Lactate	mmol/l	1.8	–	–	–	–	–	0.5-2.2
Total cholesterol	mg/dl	165	145	158	–	210	225	<200
HDL	mg/dl	66	56	68	–	58	67	>50
LDL	mg/dl	86	63	81	–	111	113	<130
Triglycerides	mg/dl	63	129	119	–	208	115	<150
Hormonal laboratory results								
Androstenedione	ng/dl	46	<30	–	–	18	<30	30-330
ACTH (8:00 a.m.)	pg/ml	<1	7.7	9.3	–	–	1.74	7.2-63.3
Cortisol (8:00 a.m.)	ug/dl	5.82	<0.05	–	–	–	0.66	5.5-25
DHEAS	ng/ml	1.7	–	2.2	2.98	–	1.5	98-340
Estradiol	pg/ml	196.3	25.7	97.8	193.9	–	44	43.9–211
FSH	uIU/ml	1.42 (follicular phase)	6.55 (follicular phase)	6.3 (follicular phase)	6.6 (follicular phase)	4.38 (follicular phase)	9.98 (follicular phase)	Follicular: 3–12
LH	uIU/ml	2.17 (follicular phase)	4.75 (follicular phase)	4.7 (follicular phase)	3.5 (follicular phase)	5.13 (follicular phase)	5.2 (follicular phase)	Follicular: 2–12; mid-cycle peak: 10–75; luteal: 1.5–10
Prolactin	ng/ml	17.58	14.07	–		–	–	4.79-23.3
SHBG	ug/dl	43.8	37.4	–		–	26	26-110
Testosterone	nmol/l	<0.09	3.0 (1 week after testosterone injection)	0.87	2.84	1.58	<0.09 (two days before testosterone injection)	0.22–2.9
17-OHP	ng/ml	1.65	0.63	0.35	0.38	0.1	0.32	Follicular: <2.0; luteal: <5.0

ALT, alanine aminotransferase; AST, aspartate aminotransferase; ALP, alkaline phosphatase; CK, creatine kinase; HDL, high-density lipoprotein; LDL, low-density lipoprotein; ACTH, adrenocorticotropic hormone; DHEAS, dehydroepiandrosterone sulfate; FSH, lollicle-stimulating hormone; LH, luteinizing hormone; SHBG, sex hormone-binding globulin; 17-OHP, 17-hydroxyprogesterone.

The patient provided detailed medical documentation from her childhood. According to these records, she was diagnosed during neonatal period with the SW form of 21OHD after experiencing a SW adrenal crisis, after which hydrocortisone and fludrocortisone therapy was initiated. Although CYP21A2 genotyping was not available, family segregation analysis based on serological HLA typing supported autosomal recessive inheritance. The patient carried two distinct HLA haplotypes, one inherited from each parent, both containing HLA-DR5, consistent with parental heterozygosity and classical salt-wasting CAH (see **Supplementary Table 1**).

From the time of the adrenal crisis throughout the entire first year of life, she was treated with hydrocortisone 17.5 mg/day and fludrocortisone 0.05 mg/day. Her growth parameters were appropriate for age, and physical examination revealed clitoromegaly and the presence of a urogenital sinus.

**Figure 1:**
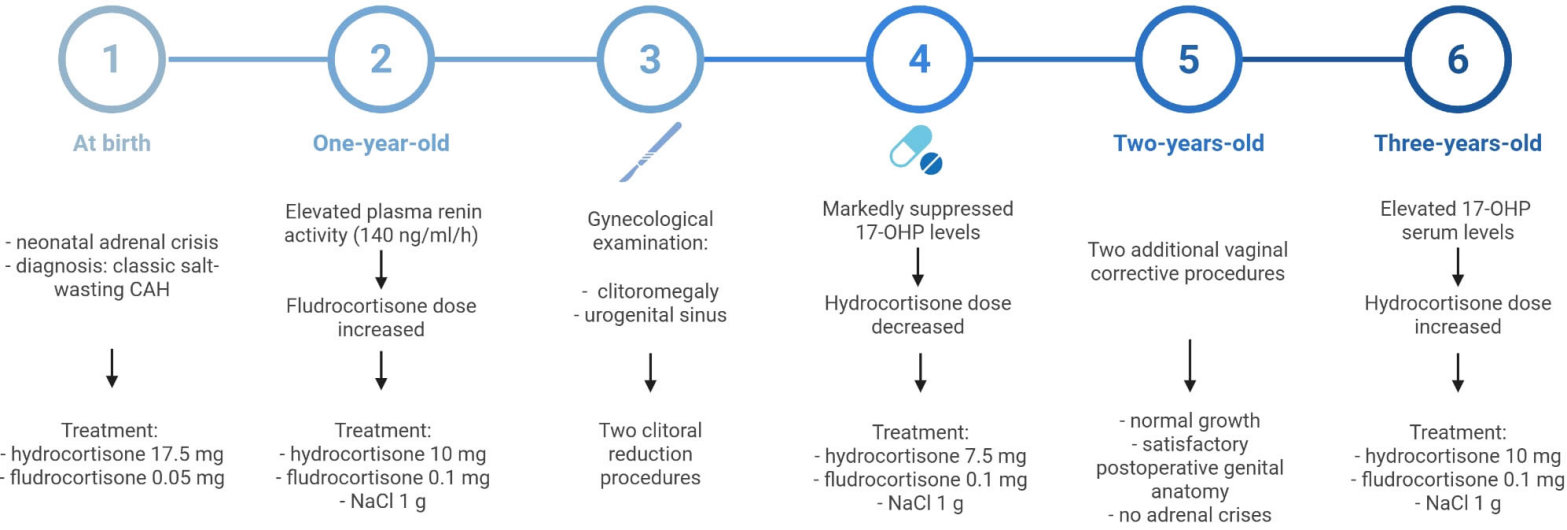
Timeline summarization of the patient’s hormonal evaluation and treatment in first years after diagnosis.

In the following year, the fludrocortisone dose was increased to 0.1 mg/day due to elevated plasma renin activity (140 ng/ml/h). Satisfactory hormonal control was achieved with hydrocortisone 10 mg/day, fludrocortisone 0.1 mg/day, and NaCl 1 g/day. Follow-up evaluation showed serum levels of 17-OHP, androstenedione (9 ng/dl), and aldosterone well below the expected physiological values, with dehydroepiandrosterone (DHEA) and testosterone remaining undetectable. The 24-hour urinary steroid profile was within expected limits, confirming effective adrenal suppression. A gynecological examination revealed clitoromegaly and the presence of a urogenital sinus. Consequently, the patient was admitted to hospital for clitoral reduction surgery, followed by further reoperation due to complications. Concurrent laboratory testing demonstrated profoundly low serum 17-OHP, which led to a subsequent decrease in hydrocortisone dosing (**Figure 1**; **Table 2**).

**Table 2:** Endocrine laboratory findings during the first three years of CAH management.

Parameter	Reference range	Unit	1 year-old	2 years-old preoperative tests	2 years-old	3 years-old
17-OHP	<1	ng/ml	2.7	0.5	3.8	15.64
DHEA	< 2.3	ng/ml	undetectable	undetectable	undetectable	Undetectable
Testosterone	< 20	nmol/l	undetectable	undetectable	undetectable	Undetectable

17-OHP, 17-hydroxyprogesterone; DHEA, dehydroepiandrosterone.

During the third year of life, the patient underwent two additional vaginal corrective procedures to optimize genital anatomy and functional outcomes. Hormonal evaluation showed low level of serum 17-OHP and persistently undetectable androgens (testosterone, DHEA) (**Table 2**). She remained on hydrocortisone 5 mg in the morning and 2.5 mg in the evening, along with fludrocortisone 0.1 mg/day. Subsequent follow-up revealed normal growth, satisfactory postoperative genital appearance, stable levels of 17-OHP and renin, and no adrenal crises. Serum androgens remained undetectable. In the subsequent year, an inspection revealed inadequate suppression of 17-OHP levels. In view of the insufficient dosage in relation to body size, the hydrocortisone dose was increased to 10 mg/day. (**Figure 1**; **Table 2**).

At the age of 28 years, the patient was switched from hydrocortisone to prednisolone 10 mg/day (administered in two daily doses) due to persistent fatigue and poor tolerability of hydrocortisone, which at that time was attributed to symptoms of adrenal insufficiency. Although no further medical documentation was available from later years, the patient reportedly continued to undergo regular follow-up evaluations and remained under the supervision of several endocrinology clinics.

The detailed analysis of the patient’s history revealed that chronic androgen suppression had been present since the neonatal period and, together with the mild Cushingoid features observed at hospital admission, suggested that the patient had been chronically overtreated with GCs. It was recognized that prolonged inhibition of the hypothalamic–pituitary–adrenal axis (HPA axis) from the neonatal period could have led to persistent adrenal hypofunction and complete androgen deficiency. Consequently, her symptoms — including muscle pain, weakness, and depressive disturbances — were attributed to androgen deficiency.

As reduction of the GC dose was not feasible due to poor tolerance, testosterone prolongatum 50 mg intramuscularly every 4–8 weeks was initiated as androgen replacement. The therapy was introduced under close clinical monitoring (after excluding contraindications such as hepatic dysfunction, dyslipidemia, or malignancy). The decision was made on an individualized, compassionate basis, with informed consent obtained from the patient, given the lack of evidence-based guidelines for androgen replacement in women with CAH.

Within three months, the patient reported marked improvement in muscle pain, strength, libido, and overall well-being. The treatment was well tolerated and continued alongside her existing prednisolone and fludrocortisone regimen. Physical examination showed increased muscle tone without signs of virilization; menstrual cycles remained regular. Serial laboratory monitoring demonstrated stable hematologic, hepatic, and renal parameters throughout follow-up. Two years after testosterone initiation, lipid profile and glycemic indices remained within normal ranges, and no evidence of hepatotoxicity or erythrocytosis. At that time point, based on follow-up hormonal assessment demonstrating supraphysiological testosterone levels one week after injection (**Table 1**), the testosterone dose was adjusted to 25 mg intramuscularly once monthly, which was subsequently maintained as the final maintenance dose for the following ten years of follow-up. During subsequent follow-up assessments at five, six and eight years after therapy initiation, hormonal profiles remained stable, with testosterone concentrations consistently within the physiological range, comparable to those observed in previous years.

At the 10-year follow-up visit, the patient reported sustained good overall well-being and absence of muscle-related symptoms. Hemoglobin and hematocrit remained within reference limits, and there were no signs of androgen excess or adverse metabolic effects (**Table 1**). Clinical evaluation revealed no evidence of androgenetic alopecia and no clinical signs of hirsutism. Follow-up DXA demonstrated maintenance of normal bone density in the lumbar spine (BMD 1.055 g/cm², T-score +0.1, Z-score +0.4) and forearm (T-score +1.1 to +1.8, Z-score 1.5 to 2.1), with only mild, localized osteopenia of the femoral neck (BMD 0.693 g/cm², T-score –1.4, Z-score 1.1). Trabecular Bone Score (TBS = 1.49) confirmed normal trabecular microarchitecture. Adrenal CT showed normally sized adrenals without nodules.

## Discussion

This case illustrates a rare but clinically relevant complication of CAH—chronic suppression of adrenal androgens most probably resulting from long-term GC overtreatment in childhood. In clinical practice, the therapeutic focus in CAH management has traditionally centered on the prevention of adrenal crises and control of androgen excess. However, prolonged GC overtreatment may lead to the opposite endocrine imbalance—functional androgen deficiency—which is rarely recognized or addressed.

In CAH due to 21OHD, standard management involves GC and mineralocorticoid replacement aimed at normalizing cortisol levels and suppressing excess androgen production (1, 5). However, achieving this balance remains challenging. Chronic GC overtreatment—often used to minimize androgen excess—can lead to iatrogenic Cushing’s syndrome, growth impairment, metabolic complications, and suppression of residual adrenal androgen synthesis - a state analogous to secondary hypogonadism. Consequently, circulating levels of testosterone, androstenedione, and DHEA decline markedly, reducing tissue androgenic activity and impairing anabolic and neurobehavioral functions. Conversely, insufficient dosing may result in androgen excess, virilization, infertility, obesity, metabolic disturbances, reduced bone mass, and impaired quality of life. While hyperandrogenism and its management dominate clinical attention, the opposite endocrine imbalance—androgen deficiency—is rarely recognized.

In the present case, ACTH, DHEAS and testosterone were profoundly suppressed, and symptoms such as myalgia, weakness, and reduced libido were attributed to severe hypoandrogenism secondary to chronic GC overtreatment. Although standard biochemical markers used to assess CAH control, including 17-OHP and androstenedione, were only moderately suppressed, this dissociation likely reflects selective impairment of adrenal androgen synthesis under long-term ACTH suppression initiated early in life, leading to chronic inhibition of the HPA axis. Notably, suppression of DHEA/DHEAS is an expected finding during GC replacement therapy in patients with CAH; however, complete suppression of circulating testosterone is not typically observed and suggests a clinically relevant functional androgen deficiency. Conventional biochemical markers offer limited insight into adrenal androgen availability, as they predominantly reflect overall disease control rather than peripheral androgen adequacy.

Introduction of low-dose testosterone replacement led to full symptomatic recovery without virilization, highlighting the physiological importance of androgens in women and the need for individualized hormonal optimization in adult CAH management. The patient’s symptom profile and favorable therapeutic response support the interpretation that functional androgen deficiency constituted a clinically meaningful consequence of long-term GC therapy in this case.

**Figure 2:**
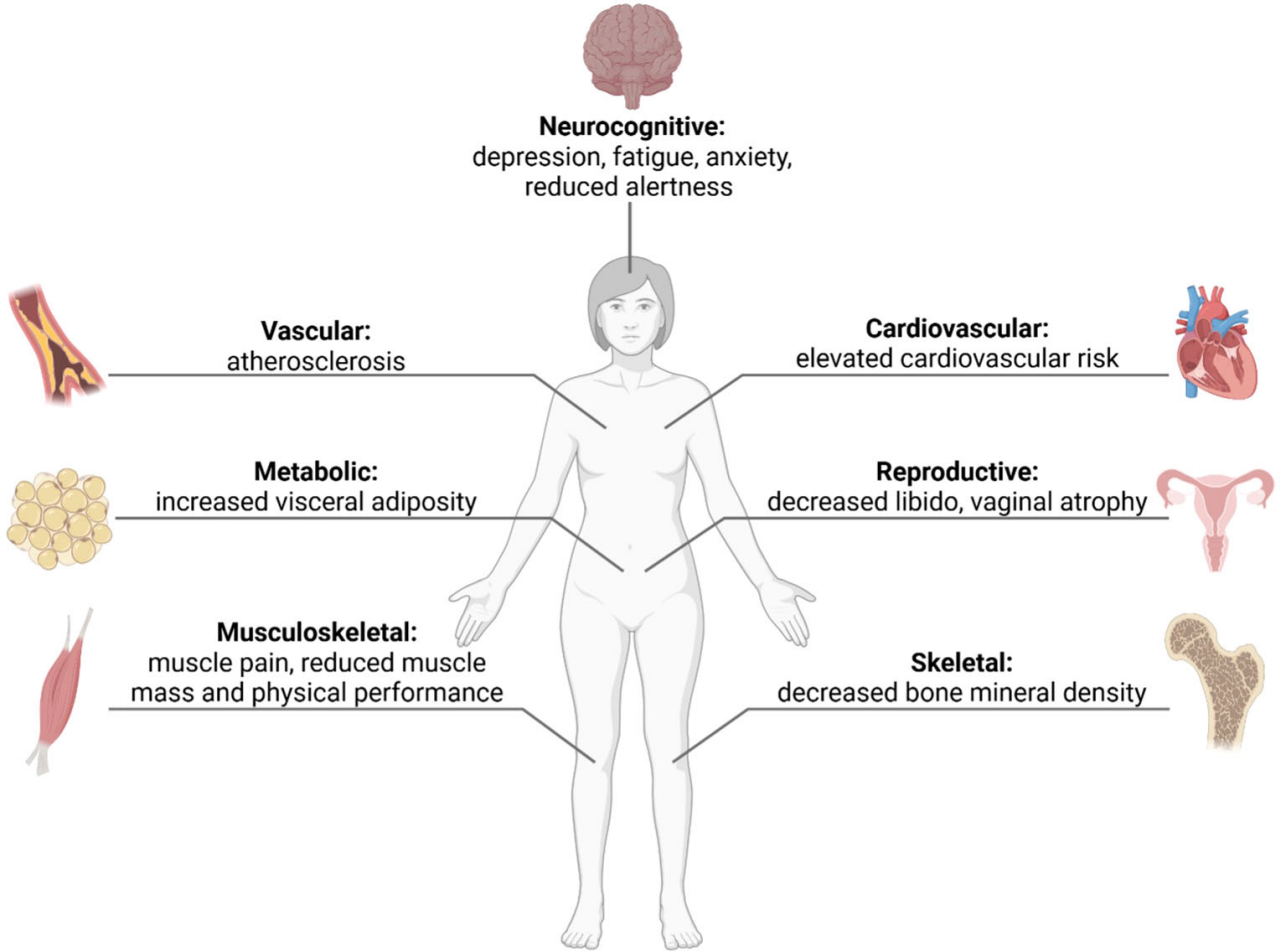
Clinical features in females with androgen deficiency.

Emerging evidence highlights the essential role of androgens in female physiology. Androgen deficiency has been associated with reduced muscle mass, chronic fatigue, mood disturbances, and sexual dysfunction (**Figure 2**)—symptoms overlapping with those of chronic GC excess. Beyond reproductive functions, testosterone and its metabolites exert anabolic, neurotrophic, and vasoprotective effects. Experimental and clinical studies have demonstrated that androgens stimulate muscle protein synthesis, satellite cell activation, and mitochondrial biogenesis, thereby improving muscular strength and endurance (6, 7).

In our case, extensive metabolic and neuromuscular work-up was unremarkable, and the clinical response to low-dose testosterone provided causal evidence for androgen deficiency as the underlying mechanism. Notably, the therapy was well tolerated and effectively prevented testosterone deficiency during follow-up, as documented two, five, six, and eight years after treatment initiation. Although only a pre-dose trough testosterone level was available at the 10-year follow-up, testosterone concentrations measured at the 8-year follow-up—during an unchanged regimen of 25 mg intramuscularly every 4 weeks maintained for six consecutive years—were within the reference range (**Table 1**). Together with the long-term absence of symptoms of androgen deficiency or excess and the sustained clinical benefit, these findings support that the administered dose achieved physiologically appropriate androgen exposure. The absence of erythrocytosis, hepatic dysfunction, or dyslipidemia during ten years of follow-up supports the safety of this approach in carefully selected cases. The favorable DXA findings demonstrated preserved skeletal integrity despite chronic GC exposure, likely reflecting the combined effect of adequate muscle mass and restored androgen activity. The absence of bone loss or structural deterioration over a decade suggests a potential protective influence of testosterone on bone microarchitecture and neuromuscular function.

Similar patterns of androgen-related symptoms have been described in other clinical contexts characterized by acquired hypoandrogenism in women. Surgical menopause after bilateral oophorectomy, treatment with GnRH agonists, long-term use of aromatase inhibitors or antiandrogenic agents have been associated with decreased muscle mass, fatigue, sexual dysfunction, and reduced quality of life (6, 8–11). Likewise, women with primary adrenal insufficiency may experience fatigue and diminished libido due to loss of adrenal androgen production, while DHEA replacement (50 mg/day) has shown modest benefits in randomized controlled trials (12). These parallels support the notion that a minimal androgen threshold is necessary for optimal physical and mental function in women.

Meta-analyses of available trials have identified no serious adverse events associated with physiological testosterone therapy in women; however, long-term safety cannot be assured, given that most studies excluded participants with significant cardiometabolic comorbidities (stroke, acute myocardial infarction, deep vein thrombosis) and were limited in duration (11, 13).

According to international consensus statements (13), testosterone therapy should only be considered after thorough evaluation, using doses that maintain serum concentrations within the normal premenopausal range. In our case, the use of low-dose intramuscular testosterone prolongatum (25 mg every four weeks) achieved physiological exposure and resulted in durable clinical improvement without virilization or menstrual disruption.

This case highlights the need to acknowledge iatrogenic androgen deficiency as a meaningful consequence of long-term CAH treatment, where the pursuit of androgen suppression can inadvertently impair muscle strength, mood, bone health, and overall functioning. Moving forward, carefully tailored androgen replacement strategies for women with CAH and other forms of acquired hypoandrogenism warrant systematic investigation, with attention to long-term safety, metabolic and functional benefits, and psychosexual well-being. Thoughtfully administered physiological androgen repletion—embedded within multidisciplinary endocrine care—holds the potential to re-establish hormonal equilibrium while enhancing patient quality of life and personal autonomy.

A limitation of this report is that at the 10-year follow-up only a pre-dose (trough) testosterone measurement was available, precluding a precise assessment of on-treatment androgen exposure at that time point. However, testosterone measurements obtained at five, six, and eight years of follow-up demonstrated stable concentrations within the laboratory reference range over an extended period, supporting sustained physiological androgen exposure. The lower testosterone concentration observed at the 10-year assessment is consistent with pre-injection sampling. Furthermore, due to the retrospective nature of the case, it was not possible to retrieve a greater number of biochemical measurements. Standardized assessments of hirsutism and androgenetic alopecia (e.g., Ferriman–Gallwey and Ludwig scales), as well as validated questionnaires assessing sexual well-being, could not be reliably obtained.

## Conclusion

Chronic GC overtreatment in women with CAH may result in complete suppression of DHEA/DHEAS and testosterone production and secondary hypoandrogenism, presenting with muscle pain, weakness, fatigue, and mood disturbances. A comprehensive, multidisciplinary approach is essential in the evaluation of chronic myalgia, as endocrine causes—particularly androgen deficiency—are often underrecognized. In carefully selected patients, low-dose testosterone replacement may represent a safe and effective therapeutic option, improving muscle function, mood, and overall quality of life, without side effects such as virilization or menstrual disturbances. Clinicians should remain alert to symptoms of androgen deficiency in adults with CAH and routinely assess androgen status as part of long-term management.

## Data Availability

The original contributions presented in the study are included in the article/**Supplementary Material**. Further inquiries can be directed to the corresponding authors.
