# Supplementary material for: Muscle pain in a woman with congenital adrenal hyperplasia due to 21-hydroxylase deficiency resolved with testosterone therapy. A case report with 10 years of follow-up
Source: Front Endocrinol (Lausanne). 2026 Feb 24;17:1757725. doi: 10.3389/fendo.2026.1757725 (PMC12971404; doi:10.3389/fendo.2026.1757725)
Supplement: Supplementary file 1 [file Table1.docx]

Supplementary Material

# Supplementary Table 1. Serological HLA typing results.

| **Individual** | **HLA haplotype 1** | **HLA haplotype 2** |
| --- | --- | --- |
| Patient | A26, Bw55(w6), Cw3, DR5(MB3, MT2) | A3, B7 (w6), Cw7, DR5(MB3, MT3) |
| Patient’s father | A26, Bw55(w6), Cw3, DR5(MB3, MT2) | Aw31, Bw51(w4), DR7(MB2, MT3) |
| Patient’s mother | A3, B7(w6), Cw7, DR5(MB3, MT2) | Aw31, Bw35(w6), Cw4, DR4 MB3, MT3) |
